# Effectiveness of Tai chi exercise on overall quality of life and its physical and psychological components among older adults: a systematic review and meta-analysis

**DOI:** 10.1590/1414-431X202010196

**Published:** 2020-09-07

**Authors:** Di Wang, Pengcheng Wang, Kun Lan, Yingchun Zhang, Yingli Pan

**Affiliations:** 1 Fourth Affiliated Hospital, China Medical University, Shenyang, China; 2 School of Nursing, Nanjing University of Chinese Medicine, Nanjing, China

**Keywords:** Tai chi exercise, Older adults, Quality of life, Physical component, Psychological component, Meta-analysis

## Abstract

With the aging of the world's population, the quality of life of older adults is becoming more important. There are many studies on the use of Tai chi exercise, a popular form of mind-body exercise practiced by older adults. However, the effectiveness of Tai chi exercise on the quality of life of older adults is unclear. For this systematic review and meta-analysis, six databases (PubMed, CENTRAL, CINAHL, EMBASE, Scopus, CNKI) were searched in English and Chinese languages to screen for relevant randomized controlled trials (RCT), and their risk of bias was assessed by two independent reviewers. The results of quality of life, physical component, and psychological component among older adults were meta-analyzed using RevMan5.3 software. The search retrieved 2577 records. After screening, a total of 10 RCTs were included in this evaluation, with a total of 1170 participants. The meta-analysis showed that compared with the control group, Tai chi exercise had a significant impact on the overall quality of life (SMD=1.23; 95%CI: 0.56–1.98; P<0.0001), and on the physical component of quality of life (MD=5.90; 95%CI: 1.05–10.75; P=0.02), but no significant impact on the psychological component of quality of life. This study had high heterogeneity. The results of this study suggest the potential use of Tai chi exercise as an activity for increased quality of life in older adults. Future research may enhance experimental rigor and explore the rationale behind Tai chi exercise.

## Introduction

In recently years, the global population has been aging rapidly. One study showed that Pacific Asia is the most rapidly aging region globally with an estimated 200 million people over 65 years of age between 2015 and 2030 (1). Due to the normal aging process and a variety of chronic diseases, older adults usually face physical and psychological health challenges. Approximately 18 million adults over 65 years old have physical limitations (2). As reported, 52% have problems in walking, grasping, handing, or pushing, and 75.3% had balance impairment (3). Physical limitations and balance disorders can lead to a decline in functional capacity of older adults, impairing their independence.

Health maintenance and physical independence of older adults have become a widely discussed topic. According to the literature, the quality of life of the elderly can be influenced by a number of factors, among which various studies emphasize the role of demographic, physical, psychological, social, and religious characteristics (4). Quality of life (QoL) is an important component in the health status of older adults.

QoL can be divided into general QoL or health-related QoL (HRQoL). The former is based on a wide range of definitions, covering well-being and happiness without reference to health problems and disorders. HRQoL is part of a multidimensional approach that takes into account physical, psychological, and social factors as well as disease-related limitations. Clinicians are paying more and more attention to exercise, which has been identified as a safe and effective way to improve aerobic capacity, strength, and HRQoL (5,6). According to the World Health Organization, participating in sports activities may play a key role in healthy aging to promote good QoL (7).

Tai chi is a traditional Chinese exercise and mind-body meditative exercise based on thousands of years of ancient history. A large number of studies have confirmed that Tai chi exercise has a positive effect on chronic diseases in older adults (8). Based on interventional studies, several systematic reviews have been performed to produce high level evidence of the effectiveness of Tai chi exercise, in terms of immunity and infections (9), cardiovascular conditions (10), metabolic syndrome (11), bone mineral density in postmenopausal women (12), sleep disorders (8), certain cancers (13), etc. Further, a systematic review and meta-analysis by Yu et al. (14) found positive effects of Tai chi exercise in psychological well-being, such as reducing stress, anxiety, depression, and mood disturbance, and increasing self-esteem. Specifically, systematic reviews and meta-analyses have been published concerning the older adult population, with conclusions that Tai chi exercise may have positive effectiveness in improving their balance function and reducing falls (15), increasing their balance confidence, and in lowering resting blood pressure.

Concerning Tai chi exercise for QoL, several randomized controlled trials have been performed, of which conclusions are inconsistent with each other to some extent (16-18). On the other hand, previous reviews show that Tai chi exercise may play some positive role in improving life status. To our knowledge, however, the limitation of such reviews is that they have not specifically focused on Tai chi exercise as in intervention for QoL, or just considered sleep quality as one of the secondary outcomes, which means low clinical significance (8). No systematic review has solely investigated Tai chi exercise as the main intervention for QoL as primary outcome in any group of population, including older adults. Therefore, we performed this systematic review and meta-analysis to explore the effectiveness of Tai chi exercise to improve QoL in older adults.

## Material and Methods

### Selection strategy

With no time limit, we performed a comprehensive search of the medical literature in 5 English databases: PubMed, Cochrane Central Register of Controlled Trials (CENTRAL), Cumulative Index of Nursing and Allied Health Literature (CINAHL), Excerpta Medica Database (EMBASE), and Scopus, which have been checked from their inception up to December, 2019. We used the following MeSH (medical subject heading) terms and text words for searching: (“Tai Ji” or “Taiji” or “Tai Ji exercise” or “Taijiquan” or “Tai Ji Quan” or “Taichi” or “Tai chi” or “Tai Chi Chuan” or “shadow boxing”) AND (“HRQoL” or “health related quality of life” or “quality of life” or “QoL” or “life quality” or “living quality”). We also searched the Chinese database “China National Knowledge Infrastructure (CNKI)”. The CNKI database was searched to ensure a more robust review because Tai chi exercise is primarily rooted in Chinese culture so many articles are published only in Chinese databases. Subject heading terms and text words included: (“太极” (Taichi) OR “太极拳” (Taichi Chuan)) AND (“生活质量” (QoL) OR “与健康相关的生活质量” (HRQoL)). Finally, a snowball search was done.

### Eligibility criteria

We included randomized controlled trials (RCTs) published from January to December, 2019, which met the following inclusion criteria: 1) studies conducted on participants who were aged 50 years and above and suffering from one or more chronic diseases or not; 2) studies evaluating Tai chi exercise interventions that were facilitated individually and/or in groups, guided by Tai chi exercise professional/non-professional, or in person and/or using audio-visual material; 3) studies that compared Tai chi exercise with standard care, waitlist control, no intervention, newspaper reading, health education, routine activity, or walking; and 4) studies that reported at least one of the outcomes of QoL using validated measurement tools. Given that Tai chi exercise is of Chinese origin, studies published in English and Chinese were included to broaden the representativeness of evidence and prevent bias.

We excluded studies that utilized exercise intervention programs with or without Tai chi exercise as a component of mixed interventions (e.g., Tai chi exercise and acupuncture). Conference proceedings, abstracts only, book chapter reviews, letters, discussions, and editorials were also excluded.

### Study selection

Search results were downloaded from the respective databases and imported into Endnote X9 software (Clarivate, USA). After removing duplicate articles, the titles and abstracts of studies were assessed independently by two authors (DW and PCW) for their relevancy to this review. Studies were then organized according to those that met, potentially met, or did not meet the eligibility criteria. The full-texts of all relevant and potentially relevant trials were retrieved and each of the two authors independently processed and analyzed the articles to determine if they were to be included in the review. A third author (YLP) was consulted for disagreements when a consensus was not established after discussion.

### Data collection process and data extraction

A modified data collection form adapted from the Cochrane data extraction form published in the Cochrane Handbook (19) for Systematic Reviews of Interventions was used to guide extraction and collection of data from the individual trials. Data extraction was carried out independently by two reviewers (DW and PCW), to minimize bias and prevent errors. A third author (YLP) was consulted to resolve discrepancies. Study authors were contacted via emails (with a maximum of three attempts) in the event of missing information. To reduce extraction error and ensure relevancy of data included, the data extraction form was piloted by testing it on two included studies. Improvements were made to the data extraction form after piloting to tailor it better to the needs of this review.

### Quality assessment

The Cochrane “Risk of Bias” assessment tool as denoted in the Cochrane Handbook of Systematic Reviews of Interventions was used to assess bias (19). The following domains were assessed; random sequence generation (selection bias), allocation concealment (selection bias), blinding of participants and personnel (performance bias), blinding of outcome assessment (detection bias), incomplete outcome data (attrition bias), and selective reporting (reporting bias).

Two reviewers (DW and PCW) independently assessed each study and made evidence-based judgements on the attempts of study authors to minimize bias in their trials. Any disagreements were discussed and resolved by a third author (YLP). Each study was methodically judged in all domains and placed into one of the following categories: low, high, or unclear risk of bias.

### Data synthesis

We synthesized extracted data using meta-analytical methods. RevMan 5.3 software, provided by the Cochrane Collaboration (UK), was used for meta-analysis. Continuous data were analyzed using the inverse variance approach by combining the mean difference of individual studies when the outcome was reported using the same measurement scale, or the standardized mean difference (SMD) of individual studies, when the outcome was reported using different measurement scales. The SMD in the meta-analysis, also known as the Cohen's d, was used to evaluate the magnitude of effect size (d<0.2, very small effect size, 0.2≤d<0.5, small effect size, 0.5≤d<0.8, moderate effect size; d≥0.8, large effect size) (19).

Heterogeneity was evaluated through computation of the I^2^ statistic and chi-squared test, with consideration for effect magnitude and direction. A chi-squared test with a P-value <0.10 (significance level of 0.1) indicated that a study was heterogeneous. The I^2^ statistic was used to assess the extent of heterogeneity (I^2^=0-30%, low; I^2^=30-60%, moderate; I^2^=50-90%, substantial, I^2^=75-100%, considerable). If heterogeneity was not significant (P-value >0.10 and I^2^<30%), the fixed-effects model was adopted. If heterogeneity was significant (P-value <0.10 and I^2^≥30%), a random-effects model was used.

## Results

### Search process

**Figure 1:**
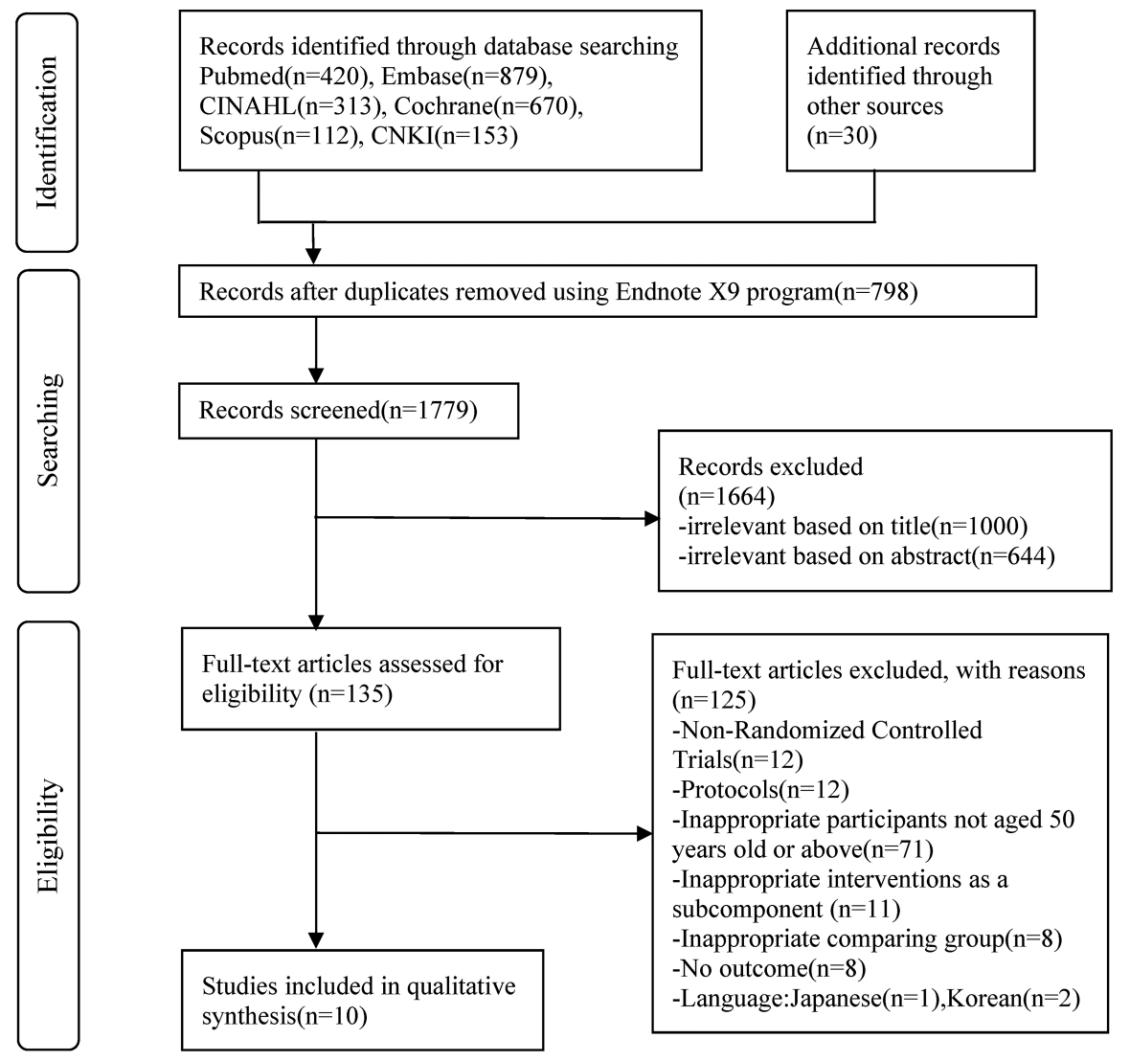
PRISMA flow diagram.

The systematic search yielded 2577 records: PubMed (n=420), EMBASE (n=879), CINAHL (n=313), Cochrane (n=670), Scopus (n=112), and CNKI (n=153). Thirty additional records were identified from other sources of reference lists. Duplicates (798) were removed, leaving 1779 records for screening. From those, 1000 records based on title and 644 records based on abstract were excluded after screening against the eligibility criteria. The full text of the remaining 135 records were retrieved, independently screened by two reviewers (DW and PCW), and 123 articles were included for the reasons as listed in Figure 1. Finally, 10 studies were eligible for inclusion in this review. The PRISMA Flowchart (Figure 1) illustrates the search process.

### Description of included studies

The data from the 10 studies are summarized in Supplementary Table S1. All studies were individual RCTs; eight were two-arm RCTs (20-27), two were three-arm RCTs (28,29). Three studies were conducted in China (21,24,26), two in Hong Kong (22,28), one in Korea (25), one in Germany (23), one in Spain (20), one in Iran (27), and one in the United States (29).

There were several types of Tai chi exercise in the 10 RCTs, including the combination of the 18 movements of Tai chi exercise (22), the 13 movements of Breathing Regulation Tai chi exercise (28), which were selected and modified from the 18 movements of Tai chi exercise produced by the Department of Health of Hong Kong, the 24 movements of Tai chi exercise (21,23,26,27), and the 20 movements of Tai chi exercise, developed by Dr. Paul Lam (see ref. 25 for details). The comparable interventions in the control groups were usual care, exercise, slow walking, traditional physical exercise, and usual daily activities.

Tai chi exercise was studied in older adults, older adults with chronic obstructive pulmonary disease, older adults admitted to an intermediate care rehabilitation unit, older adults with congestive heart failure and depression, older adults with benign prostate hyperplasia, elderly females with low bone mass, highly maladjusted institutionalized elderly, and older adults with total knee arthroplasty. The length of the Tai chi exercise interventions ranged from 4 weeks to 24 weeks, with a median duration of 14.4 weeks for studies assessing HRQoL. The duration of the individual sessions ranged from 15 to 90 min, frequency from two to five times per week.

To evaluate HRQoL outcomes, the following instruments were used: Medical Outcomes 12-item Short Form Health Survey (SF-12), St. George's Respiratory Questionnaire Hong Kong Chinese (SGRQ-HKC), a visual analogical scale (VAS), Minnesota Living with Heart Failure Questionnaire (MLHF), Chinese version of WHOQOL-BREF, Urination-Related Quality of Life (QoL), Medical Outcomes 36-item Short Form Health Survey (SF-36), Activities of Daily Living (ADL), Neuropsychiatric Inventory(NPI), and Leiden Padua Quality of Life questionnaire (LEIPAD).

### Risk of bias

Table 1 presents the risk of bias summary of the included studies. Eight studies detailed their random sequence generation process (20-23, 25-28) and six studies described allocation concealment measures (20,22,23,26-28). Due to the nature of Tai chi exercise intervention, it was difficult to blind participants, resulting in only two studies describing the blinding of participants and personnel, and the others obtaining either an unclear or high risk of performance bias (20,28). Three studies reported blinding of outcome assessors (22,25,27). All studies had a low incomplete outcome data bias, a low selective outcome reporting bias, and a low other sources of bias (20-29).

**Table 1:** Risk of bias assessment of included randomized clinical trials.

	Random sequence generation	Allocation concealment	Blinding of participants and personnel	Blinding of outcome assessment	Incomplete outcome data	Selective outcome reporting	Other sources of bias	Total
Chan et al. 2017 (22)	+	+	−	+	+	+	+	6
Chan et al. 2010 (28)	+	+	+	?	+	+	+	6
Martinez et al. 2015 (20)	+	+	+	+	+	+	+	7
Yuan et al. 2016 (21)	+	−	?	−	+	+	+	4
Cui et al. 2017 (24)	?	−	−	?	+	+	+	3
Jung et al. 2012 (25)	+	−	−	+	+	+	+	4
Chyu et al. 2010 (23)	+	+	?	−	+	+	+	5
Dechamps et al. 2010 (29)	?	−	?	?	+	+	+	3
Li et al. 2019 (26)	+	+	?	−	+	+	+	5
Tajik et al. 2018 (27)	+	+	−	+	+	+	+	6

+: Low risk of bias; ?: Unclear risk of bias; -: High risk of bias (mean score for risk of bias was 4.9).

### Effectiveness of Tai chi exercise on overall QoL

**Figure 2:**
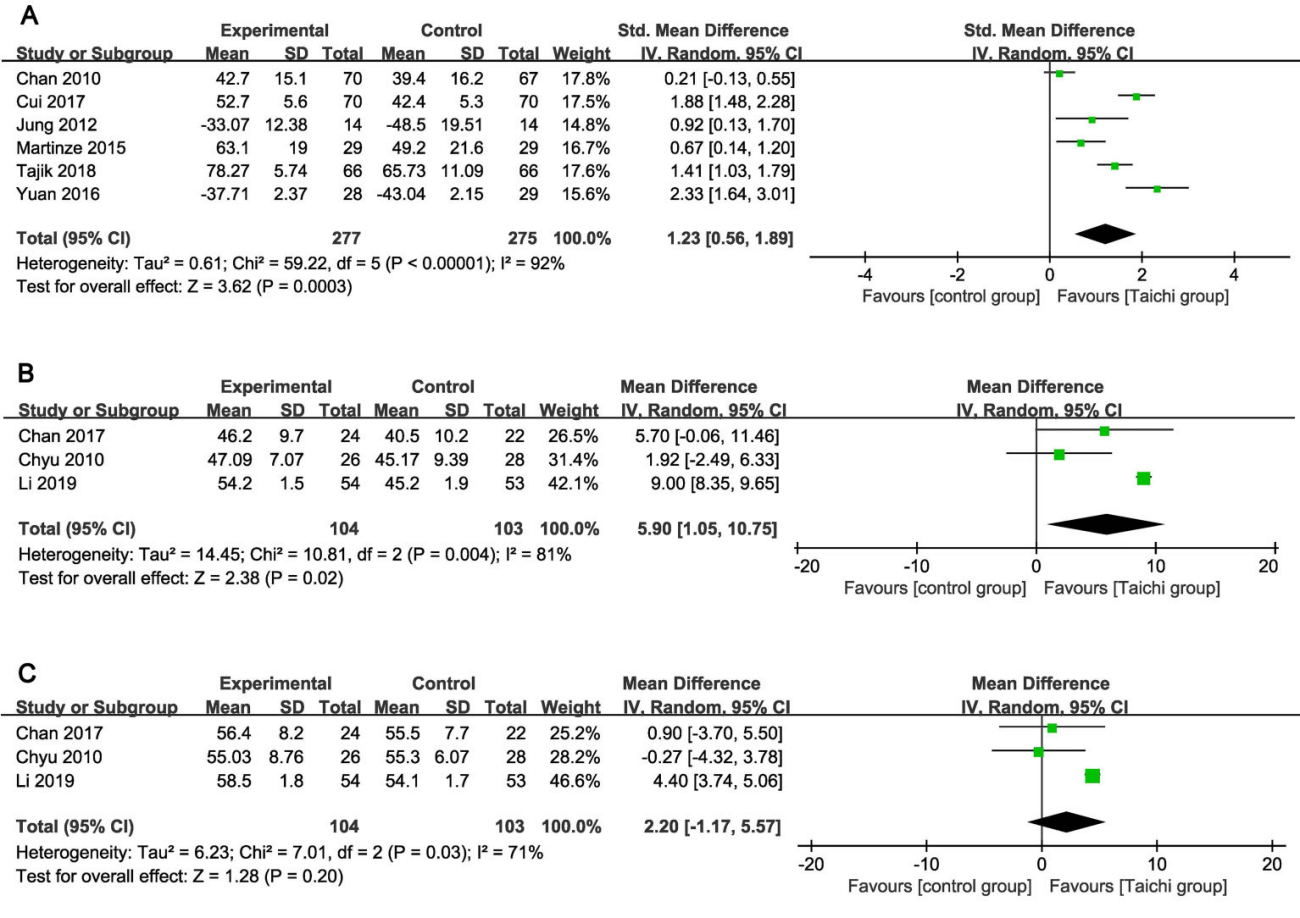
Forest plot of the effectiveness of Tai chi exercise on quality of life in older adults. See Table 1 for reference numbers.

We performed the meta-analysis on overall QoL. Different scales were used in the six studies to measure and evaluate the QoL. In four of the studies (20,24,27,28), the higher the score, the better the QoL, while in the other two studies (21,25), the higher the score, the worse the QoL. For the two studies (21,25), we took the mean times as described in the Cochrane Handbook (16) and converted these values into the new value, with the standard deviation unchanged. Six studies involving 277 participants in the Tai chi exercise group and 275 participants in the control group were meta-analyzed for overall QoL. The pooled results showed statistically significant effect favoring Tai chi exercise (SMD=1.23; 95%CI: 0.56-1.98; P<0.0001). Significant and substantial heterogeneity was found among the combined studies (I^2^=92%; P=0.0003) (Figure 2A).

### Effectiveness of Tai chi exercise on physical component and psychological component of QoL

Three studies involving 104 participants in the Tai chi exercise group and 103 participants in the control group were meta-analyzed for the physical component and the psychological component (22,23,26). The Tai chi exercise group showed better performance on the physical component (MD=5.90; 95%CI: 1.05-10.75; I^2^=81%; P=0.02) compared with the control group (Figure 2B). When combining all three RCTs in pool analysis, the direction on the mental component favored the Tai chi exercise compared with the controls, but since the 95% confidence interval included zero, the difference was not significant (MD=2.20; 95%CI: -1.17-5.57; I^2^=71%) (Figure 2C).

### Sensitivity analysis

#### Sensitivity analysis on overall QoL

The fixed effect model was used to recombine the statistics. The Tai chi exercise group was statistically significant in improving QoL (SMD=1.10; 95%CI: 0.91-1.28; P<0.01). This was the same as the results of the random effects model, indicating that the results were stable.

#### Sensitivity analysis on physical component

The fixed effect model was used to recombine the statistics. The Tai chi exercise group was statistically significant in the physical component (MD=8.81; 95%CI: 8.17-9.45; P<0.01). This was the same as the results of the random effects model, indicating that the results were stable.

## Discussion

This review aimed to examine the effectiveness of Tai chi exercise on the QoL among adults aged 50 and above with or without chronic diseases. The 10 RTCs studies involved 1030 participants. The duration of interventions ranged from four weeks to twenty-four weeks with varying forms of Tai chi exercise.

Six studies that measured overall QoL using the correlation scales were meta-analyzed revealing a positive effect of Tai chi exercise (20,21,24,25,27,28). Although the analysis showed high heterogeneity, the sensitivity analysis showed the same efficacy. All six studies used different tools to measure self-efficacy, which may have led to the high degree of heterogeneity. This result is consistent with a recent review on Tai chi exercise for women with cancer that also reported significant improvements in QoL with Tai chi (30). Chronic disease compromises QoL by slowly decreasing life control and the ability to participate in meaningful activities and pursue desired outcomes. All participants in the assessment were over 50 years old, which meant they may lack control in their lives. One plausible reason for the current findings is that participants who completed Tai chi exercise interventions regained personal sensory control, which is an important determinant of QoL in older adults, increasing their confidence in disease management and improving people's overall quality of life.

The meta-analysis of three studies showed a good effect in improving physical components, which are part of QoL. Significant heterogeneity was detected, which may be related to diverse forms and durations of Tai chi interventions and different health conditions among older adults.

Although the difference was not statistically significant, more than 50% of individual RCTs reported that Tai chi exercise improved psychological components related to QoL. Other similar reviews found that psychological components such as depression were significantly reduced in studies comparing Tai chi exercise to passive controls (15,31). The possible reason for the difference in the significance of the results may be the differences in the studies included in the evaluation. Further high-quality studies are needed to develop conclusive evidence and reconcile the differences on Tai chi exercise in alleviating psychological components in older adults.

### Limitations

There are several limitations to this systematic review. Most studies were conducted in Asia and the review was limited to studies in English and Chinese languages. The limited number of RCTs and the heterogeneity in outcome measurements, Tai chi exercise interventions, intervention durations, frequency of weekly class, and methodological rigor made it difficult to determine the effectiveness of Tai chi exercise on QoL outcomes in older adults and to recommend minimum effective dosage, types, intensity, or format (sitting, standing). In addition, trials of complementary therapies published outside mainstream journals may be biased systematically.

### Implications for practice and research

Tai chi exercise is a safe, economical, and beneficial exercise that is easy to learn. According to this systematic review, Tai chi exercise can be considered a sport to improve the QoL of older adults. However, before recommending Tai chi exercise, healthcare professionals should make different recommendations based on the situation and diagnosis of older adults. Future randomized controlled trials should provide more rigorous research procedures, in particular measures to reduce bias, so that more accurate assessments of quality and intervention effectiveness can be achieved.

### Conclusions

This systematic review demonstrated that Tai chi may be an exercise option for older adults, with a positive effect on overall QoL and physical component. However, for the psychological component, the effect was not significant.

## Supplementary Material

Click here to view [pdf].
